# Research on automatic pilot repetition generation method based on deep reinforcement learning

**DOI:** 10.3389/fnbot.2023.1285831

**Published:** 2023-10-11

**Authors:** Weijun Pan, Peiyuan Jiang, Yukun Li, Zhuang Wang, Junxiang Huang

**Affiliations:** ^1^Air Traffic Control Automation Laboratory, College of Air Traffic Management, Civil Aviation Flight University of China, Deyang, China; ^2^Department of Safety Management, Xiamen Air Traffic Management Station, East China Air Traffic Management Bureau, Xiamen, China

**Keywords:** controller training, transfer learning, text generation, reinforcement learning, generalization

## Abstract

Using computers to replace pilot seats in air traffic control (ATC) simulators is an effective way to improve controller training efficiency and reduce training costs. To achieve this, we propose a deep reinforcement learning model, RoBERTa-RL (RoBERTa with Reinforcement Learning), for generating pilot repetitions. RoBERTa-RL is based on the pre-trained language model RoBERTa and is optimized through transfer learning and reinforcement learning. Transfer learning is used to address the issue of scarce data in the ATC domain, while reinforcement learning algorithms are employed to optimize the RoBERTa model and overcome the limitations in model generalization caused by transfer learning. We selected a real-world area control dataset as the target task training and testing dataset, and a tower control dataset generated based on civil aviation radio land-air communication rules as the test dataset for evaluating model generalization. In terms of the ROUGE evaluation metrics, RoBERTa-RL achieved significant results on the area control dataset with ROUGE-1, ROUGE-2, and ROUGE-L scores of 0.9962, 0.992, and 0.996, respectively. On the tower control dataset, the scores were 0.982, 0.954, and 0.982, respectively. To overcome the limitations of ROUGE in this field, we conducted a detailed evaluation of the proposed model architecture using keyword-based evaluation criteria for the generated repetition instructions. This evaluation criterion calculates various keyword-based metrics based on the segmented results of the repetition instruction text. In the keyword-based evaluation criteria, the constructed model achieved an overall accuracy of 98.8% on the area control dataset and 81.8% on the tower control dataset. In terms of generalization, RoBERTa-RL improved accuracy by 56% compared to the model before improvement and achieved a 47.5% improvement compared to various comparative models. These results indicate that employing reinforcement learning strategies to enhance deep learning algorithms can effectively mitigate the issue of poor generalization in text generation tasks, and this approach holds promise for future application in other related domains.

## 1. Introduction

**Figure 1:**
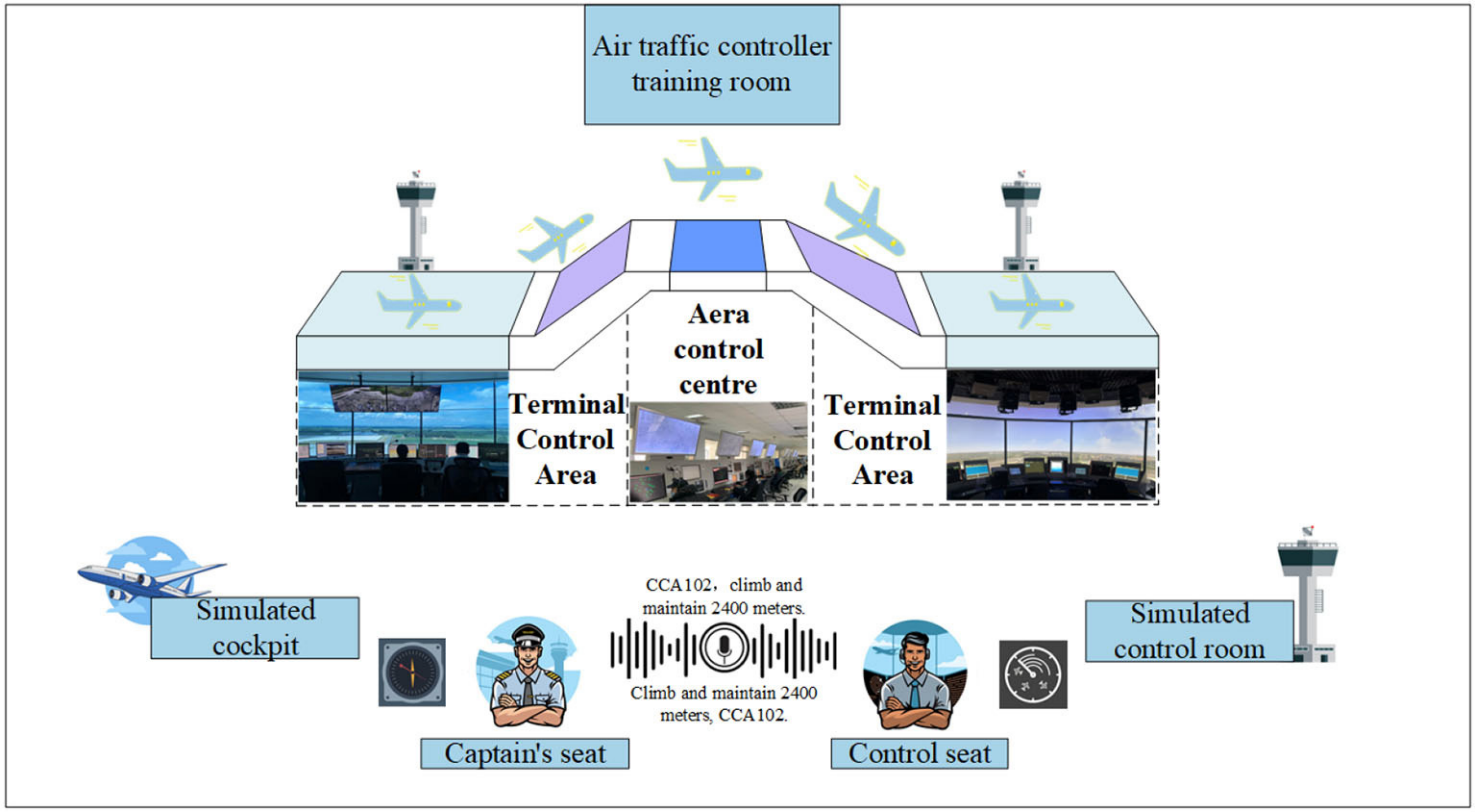
ATCOs training process.

**Figure 2:**
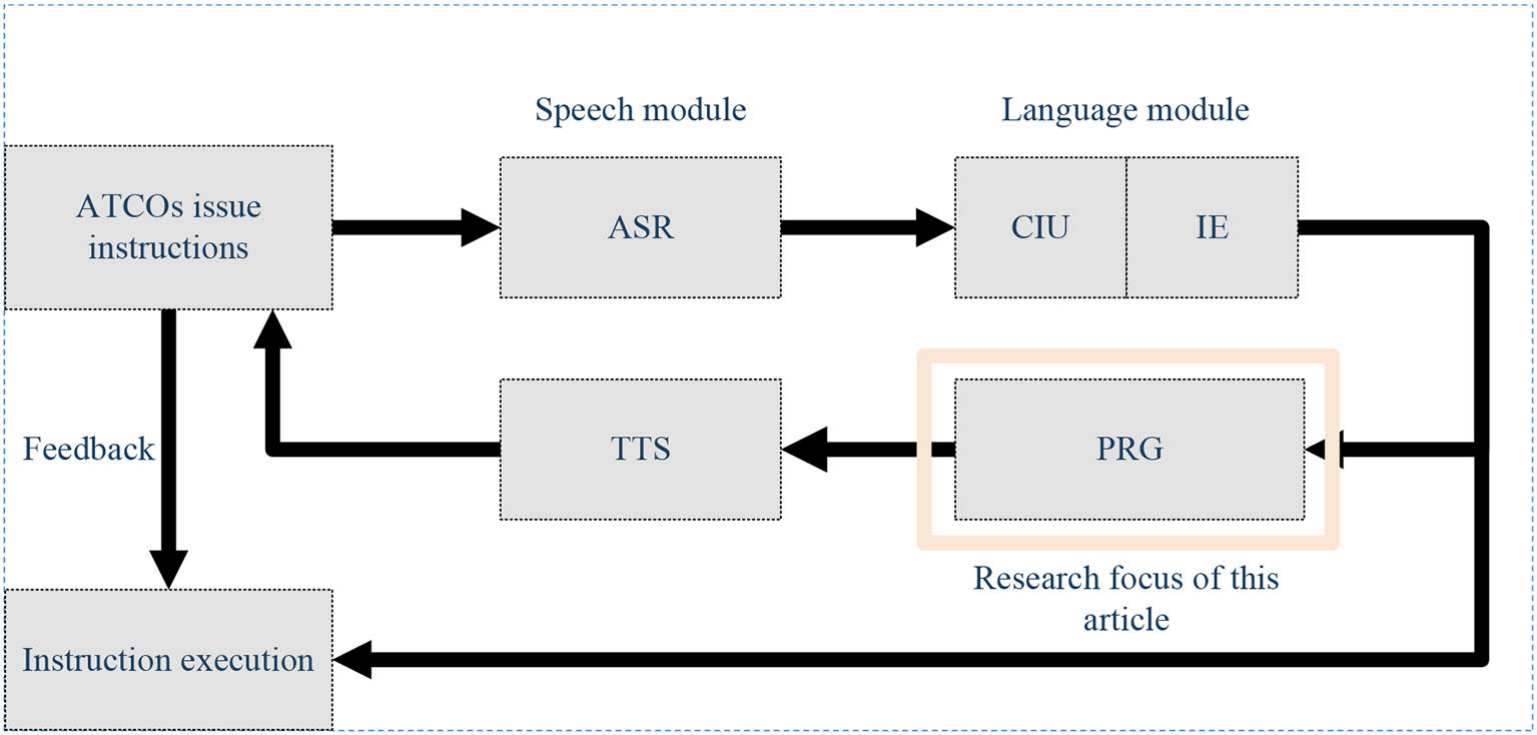
Core technologies of automatic pilot seat.

In recent research projects (Holone and Nguyen, 2015) and as indicated by the International Civil Aviation Organization (ICAO), it is projected that air traffic flow will continue to grow at an annual rate of 3 to 6% after 2025. Consequently, the demand for Air Traffic Controllers (ATCOs) will increase year by year. ATCOs communicate control instructions to pilots via Very High-Frequency (VHF) radio to manage air traffic. According to safety and reliability regulations in Air Traffic Control (ATC), pilots are required to promptly and accurately repeat control instructions they receive to ensure the correct understanding of instructions issued by ATCOs (Lin et al., 2019). ATCOs undergo specific training, including foundational courses and simulator training, to qualify for working in actual ATC scenarios. Control training simulators typically consist of two seats: one for the controller and the other for the pilot. Completing controller training requires dedicated personnel to control the pilot seat for the repetition and response to control instructions, incurring additional training costs, including equipment and personnel expenses, as illustrated in Figure 1 (Zhang et al., 2022a). In recent years, artificial intelligence (AI) technologies have been widely applied in the ATC domain (Lin, 2015; Srinivasamurthy et al., 2017; Yang et al., 2019). To alleviate the workload of ATCOs, the European Union (EU) has introduced Automatic Speech Recognition (ASR) technology into ATC to reduce their workload (Helmke et al., 2016) and enhance work efficiency (Helmke et al., 2017). Projects funded by Horizon 2020 have also constructed ATCO decision support systems using AI technology to alleviate the workload of ATCOs (Kleinert et al., 2017). These research endeavors aim to assist controllers with intelligent systems to reduce error rates and alleviate workload. Furthermore, enhancing the quality of ATCO training is another approach to reducing potential human errors (Yiu et al., 2021). Some scholars have explored the use of intelligent systems to improve the training efficiency and professionalism of ATCOs, fundamentally reducing human errors. For example, Hoekstra and Ellerbroek (2016) developed an ATC simulator called “BlueSky,” which significantly advanced research in air traffic management (ATM) despite its lower level of intelligence. Lin et al. (2021) proposed an AI-based pilot framework for ATCO training, capable of replacing the pilot seat with relatively high confidence. This framework covers several core technologies, including speech recognition, Controlling Instruction Understanding (CIU), Information Extraction (IE), Pilot Repetition Generation (PRG), Text-to-Speech (TTS), and human-computer interaction technology, as illustrated in Figure 2. Zuluaga-Gomez et al. integrated various state-of-the-art AI-based tools to build an automatic captain system, expediting the training process for air traffic controllers (ATCo) (Zuluaga-Gomez et al., 2023). However, the above research primarily focuses on the entire pilot system, with limited in-depth research on the PRG module. Building upon the aforementioned research efforts, this paper delves deeper into the task of PRG and presents novel advancements.

In Figure 1, Area Control Centers (ACC) are responsible for managing the airspace within a designated region, coordinating aircraft flights, and ensuring the orderly flow of air traffic and the tower primarily oversees the Terminal Control Area (TMA), which encompasses the airspace including airports and their surrounding regions. Due to the differences in the scope of controlled airspace, there are significant variations in the content of control instructions, leading to disparities in the data distributions between the two.

The focus of this study is on the PRG, which belongs to the field of Natural Language Processing (NLP) and falls under the task of Natural Language Generation (NLG). We achieved PRG by fine-tuning pre-trained language models based on Transformer and Seq2Seq architectures. Furthermore, we employed the policy gradient algorithm from reinforcement learning to further optimize the model and overcome the issue of poor generalization in transfer learning. The innovations of this paper are as follows: (1) Addressing the characteristics of pilot repetition generation tasks, we transformed the human-machine dialogue problem into a text summarization problem, providing a new perspective for related research. (2) By utilizing transfer learning strategies, we overcame the limitations of insufficient training data in this field, caused by the difficulty of data collection. (3) We used the policy gradient algorithm to optimize the cross-entropy loss function, overcoming the exposure bias issue associated with using cross-entropy loss in text generation tasks and enhancing the generalization of the transfer learning model. (4) We constructed a control instruction text dictionary based on the structural features of control instruction texts. This dictionary enables fine-grained tokenization of control instruction texts, facilitating subsequent metric evaluations. In addition, based on control instruction tokenization, we introduced a keyword-based evaluation to assess the quality of generated pilot repetitions. The introduced keyword evaluation metrics provide an intuitive reflection of the model's performance.

## 2. Related work

The general characteristics of PRG are as follows: (1) The length of the repetition instructions is generally shorter than that of the control instructions, and for mandatory control instructions, the repetition instructions should be consistent with the meaning of the control instructions. (2) There are fewer instances of ongoing dialogues (similar to single-turn dialogues in human-machine conversations). Based on these characteristics, PRG can be transformed from a human-machine dialogue task to a text summarization task for processing. Currently, text summarization techniques can be classified into extractive summarization and abstractive summarization based on the summarization method (Nazari and Mahdavi, 2019). Extractive summarization extracts keywords based on their importance and forms a summary. However, it only considers the word frequency and does not take into account the semantic information of sentences, resulting in poor coherence of the generated sentences. On the other hand, abstractive summarization summarizes the essential information of sentences through paraphrasing and synonym replacement. Compared to extractive summarization, abstractive summarization has better representation ability and can understand the contextual semantics of sentences. In the task of automatic text summarization, since both the input and output are text sequences, the model needs to pay more attention to the relationship between the semantic information of generated sentences and the coherence of sentences (Liu et al., 2021).

Over the years, the development of automatic text summarization has been slow due to the limitations of statistical-based methods in text representation, understanding, and generation capabilities (Zhang et al., 2019). Recently, with the continuous improvement of neural network theory and technology, deep learning has emerged as one of the most promising approaches and has achieved state-of-the-art results in many tasks (de Souza et al., 2018; Luo et al., 2019; Mane et al., 2020; Miao et al., 2020). Among them, the introduction of automatic text summarization models based on the encoder-decoder architecture has brought new advancements to deep learning-based automatic text summarization (Zhang et al., 2022b). In the current context, with the advancement of sequence-to-sequence frameworks, generative models tend to outperform extractive models (Alexandr et al., 2021).

Most of the research on generative summarization focuses on the encoder-decoder structure of sequence-to-sequence models, addressing various issues in the summarization process by incorporating attention mechanisms, pointer-generator mechanisms, coverage mechanisms, or replacing recurrent neural networks (RNNs) with convolutional neural networks. Rush et al. (2015) were the first to use attention mechanisms on the seq2seq model to address headline generation. To further improve model performance, Nallapati et al. proposed the pointer generator model (Nallapati et al., 2016b), which successfully handles out-of-vocabulary (OOV) words due to limited vocabulary. This model was later improved with the use of coverage mechanisms (See et al., 2017). Since the encoder and decoder in the Seq2Seq architecture are implemented using convolutional neural networks or RNNs, their feature extraction capabilities are not as powerful as the Transformer model. The emergence of the Transformer model based on self-attention architecture has ushered in a new era in NLP, ensuring that models can learn deeper language logic and semantic information of words. Examples of such models include BERT (Devlin et al., 2018), GPT-2 (Radford et al., 2019), Bart, and Roberta. BERT predicts words based on their contextual information, while GPT-2 predicts words based on the preceding context. Therefore, BERT is suitable for natural language understanding (NLU) tasks, while GPT-2 is more suitable for NLG tasks. Inspired by BERT and GPT-2, the Bart model combines the strengths of both, making it more suitable for text generation scenarios compared to BERT and achieving better results than GPT-2 (Lewis et al., 2019). The RoBERTa model (Liu et al., 2019), compared to BERT, GPT-2, and Bart, has advantages in terms of pre-training methods, deeper network structure, larger batch size, and unmasked training, especially for text summarization tasks. These advantages enable RoBERTa to better understand semantics, capture language features, and generate more accurate and coherent text summaries. The proposed deep reinforcement learning model in this paper is based on RoBERTa.

## 3. Challenges in PRG and our work

### 3.1. Challenges in PRG

(1) With the increase in the number of parameters in deep learning models, training high-performance models in supervised learning requires a large amount of data. In the field of ATC, data acquisition is extremely challenging due to the confidentiality of the data. Additionally, the obtained raw ATC voice data needs to be professionally annotated, which incurs high annotation costs. These factors pose significant challenges to the application and development of deep learning techniques in this domain. (2) Current NLG models often suffer from poor generalization, and this issue becomes more pronounced in the case of small datasets. Improving model generalization is a challenging task that requires extensive research. (3) Since control instructions are composed of a series of keywords (Pan et al., 2023), evaluating the generated pilot repetition instructions using ROUGE-N and ROUGE-L standards requires the segmentation of the control instructions. This necessitates the construction of a dictionary, adding extra workload. Furthermore, the specific nature of pilot repetition instructions limits the effectiveness of using ROUGE-N and ROUGE-L for evaluating the quality of generated instructions. Therefore, a new evaluation metric is needed to assess the quality of generated pilot repetition instructions.

### 3.2. Our work

We have conducted in-depth research on text generation. We found that NLG involves three major tasks: neural machine translation (NMT), text summarization, and dialogue response generation (Nallapati et al., 2016a). These tasks share the common characteristic of having text sequences as inputs and outputs, but they also have differences. The difference between text summarization and machine translation lies in the fact that generated summaries are typically very short and not influenced by the length of the source text, while the generated summary and the source text need to be semantically consistent (Zhou, 2012). Furthermore, text summarization involves compressing the source text in a lossy manner while retaining key information, which contradicts the lossless requirement of machine translation (Hastie, 2012). The difference between dialogue response generation and text summarization is that the generated text in dialogue response has logical coherence with its preceding and following context. Currently, there is no unified evaluation criterion for the quality of dialogue generation results (Song et al., 2019). PRG is a special NLG task that belongs to both dialogue response generation and text generation tasks. For certain inquiry instructions (such as “please respond when received”), the nature of their repetition belongs to dialogue, with logical relationships between the preceding and following text. However, most control instructions are mandatory instructions, and the nature of their repetition belongs to text summarization, where the meaning should remain consistent throughout.

Based on the analysis of PRG tasks mentioned above, we have adopted the following strategies from the perspective of text summarization to address the challenges faced by repetition generation. For challenge one, we use transfer learning by pretraining the model on other domain data and fine-tuning it on the target domain to achieve the generation of repetition instructions. For challenge two, we employ the policy gradient algorithm from reinforcement learning to optimize the cross-entropy loss in the pre-trained model. The cross-entropy loss relies on target labels in the training data for parameter optimization. This leads to a significant decrease in model performance when applying the fine-tuned model to similar datasets due to differences in the training label distribution. The core of the policy gradient algorithm is to optimize the parameters of the policy network by evaluating the quality of generated summaries. This allows the model to learn how to generate high-quality summaries rather than generating text summaries similar to the training sample labels, greatly improving the generalization performance of the transfer learning model. Additionally, we compare the effects of fine-tuning current mainstream pre-trained models to demonstrate the effectiveness of our proposed model. For challenge three, to enable a detailed evaluation of model performance and facilitate model improvement, we use a new evaluation criterion to assess the quality of generated repetition instructions. This criterion provides a more accurate reflection of the model's performance compared to the ROUGE evaluation criterion. Furthermore, we construct a control instruction text dictionary based on the control instruction text dataset. Using the Jieba word segmentation tool, we split the generated instruction text based on coarse-grained and fine-grained information, allowing the calculation of various metrics using computer programs.

## 4. Methodology

### 4.1. Proposed framework

Deep Reinforcement Learning (DRL) is a method that combines deep learning and reinforcement learning to solve decision-making problems with high-dimensional state and action spaces. It uses deep neural networks (DNNs) as function approximators to learn value functions or policy functions, enabling end-to-end learning from raw input to action selection. In text summarization tasks, DRL can be used to train models to generate high-quality summaries (Keneshloo et al., 2019; Sun et al., 2021). The application of DRL in text summarization generally follows the basic framework of reinforcement learning. In this framework, an agent learns the optimal policy by interacting with the environment. In this case, the environment consists of the original text and the generated summary, and the agent observes the current text state and selects actions to generate the next word. The reward function provides rewards to the agent based on the quality evaluation of the generated text, with higher rewards indicating higher-quality summaries. The key to applying DRL in text summarization lies in designing appropriate state representations, action spaces, reward functions, and policy networks. State representation refers to transforming the original text into continuous vector representations using word embeddings or encoder networks to capture the semantic and contextual information of the text. The action space defines the operations that the agent can choose, typically selecting the next word to generate from a vocabulary. The reward function is used to evaluate the quality of the generated summary. Language model-based metrics such as ROUGE evaluation can be used as the reward function to measure the similarity between the generated summary and the reference summary. The policy network is a DNN that selects actions to generate the next word based on the current state. RNNs or attention mechanisms can be used to capture the context of the text and make sequential word decisions. By applying DRL to text summarization, the model can learn to generate high-quality summaries through interactions with the environment. During the training process, the agent optimizes the parameters of the policy network to maximize the cumulative reward while generating summaries. This approach allows for end-to-end training on large-scale datasets without the need for manual annotations, leveraging deep learning techniques to extract features from raw input and generate more accurate and fluent summaries.

**Figure 3:**
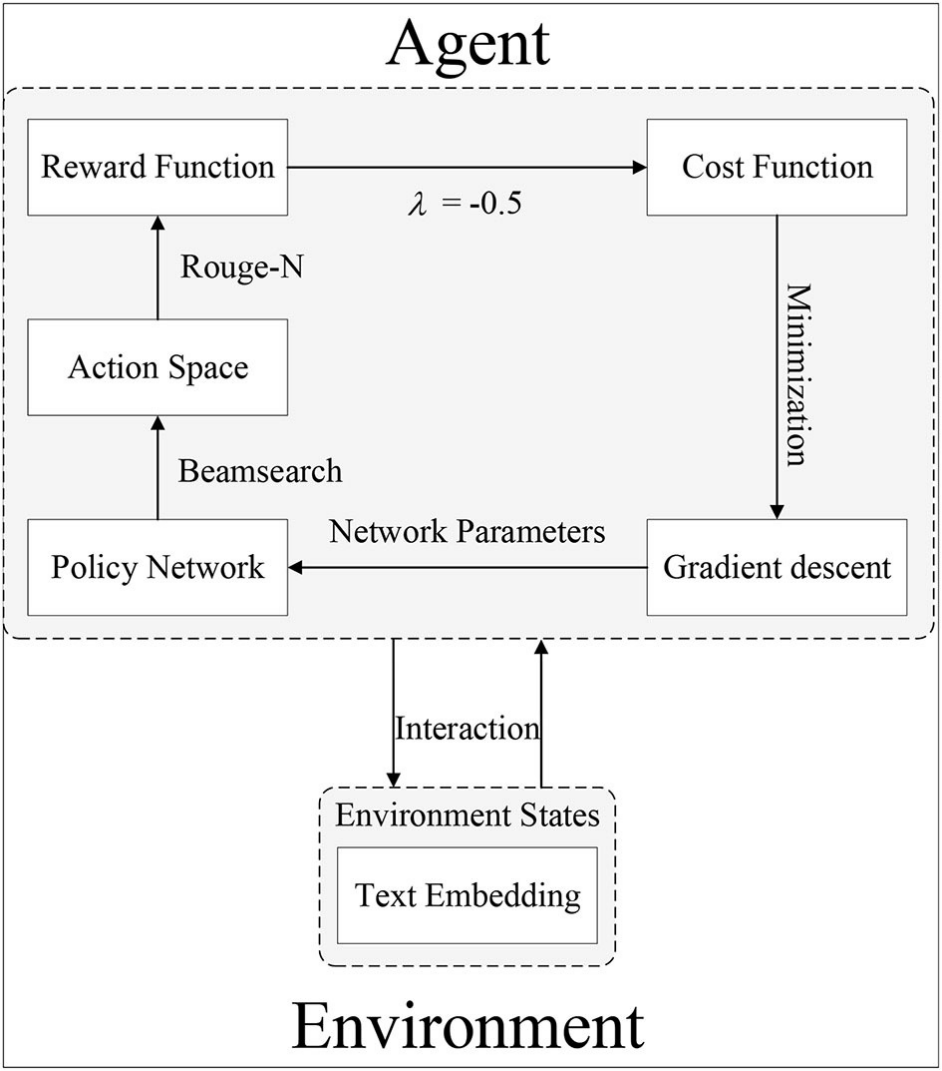
The proposed DRL architecture.

In our proposed RoBERTa-RL model, we use Word Piece embedding as the state representation of the environment. We use ROUGE-1 as the reward function and RoBERTa as the policy network. The action generation policy is implemented using Beam Search, and parameter updates are performed using the policy gradient algorithm. The architecture of our proposed deep reinforcement learning model, RoBERTa-RL, is illustrated in Figure 3.

### 4.2. Training process of RoBERTa-RL

Figure 3 provides a detailed description of the training process of the proposed DRL model architecture. Let's assume *S* = {*x*_1_, *x*_2_, ..., *x*_*n*_} represents the original input text, where *x*_1_, *x*_2_, ..., *x*_*n*_ are input characters. Firstly, *S* undergoes RoBERTa encoding to convert it into the state representation of the environment, denoted as *h*_*t*_. This process is described by Equation (1), where *RoBERTa*_embedding_() represents the encoding function:


(1) $$\begin{array}{l} h_{t}=RoBERTa_{\text{embedding}}\left(S\right) \end{array}$$

The policy network generates the output text *y*_*t*_ based on the state representation *h*_*t*_ of the input environment and the action policy Beam search. The specific process is described by Equation (2), where *Beamsearch*() represents the action policy function:


(2) $$\begin{array}{l} y_{t}=Beamsearch\left(RoBERTa,h_{t}\right) \end{array}$$

The ROUGE function calculates the reward value *R*_*t*_ based on the generated text *y*_*t*_ and the reference summary *T*_reference_. The specific formula is described by Equation (3), where *ROUGE* − 1() represents the reward function.


(3) $$\begin{array}{l} R_{t}=ROUGE-1\left(y_{t},T_{\text{reference}}\right) \end{array}$$

The cost function *COST* is composed of the weighted sum of the negative average reward value and the cross-entropy loss, where λ is the weight. The specific formula is described by Equation (4).


(4) $$\begin{array}{l} COST=-\lambda mean\left(R_{t}\right)+\left(1-\lambda \right)CrossEntorpyLoss\left(y_{t},T_{\text{reference}}\right) \end{array}$$

The policy update is performed using the policy gradient algorithm, which updates the policy network parameters θ based on the gradient of the cost function. The specific formula is described by Equation (5), where α represents the learning rate.


(5) $$\begin{array}{l} \theta =\theta -\alpha \nabla \theta \end{array}$$

### 4.3. Evaluation criteria

ROUGE (recall-oriented understudy for gisting evaluation) measures the quality of summaries by calculating the overlap units (such as n-grams, word sequences, and word pairs) between the generated summary and the reference summary (Lin and Och, 2004; Elmadani et al., 2020). This evaluation criterion has been widely used for evaluating automatic summarization tasks. ROUGE-1 and ROUGE-2 are used to assess informativeness, while ROUGE-L is used to assess fluency. N is typically set to 1 or 2. The ROUGE-1 and ROUGE-2 scores have been shown to be the most consistent with human judgments. The calculation method for ROUGE-N is described by Equation (6).


(6) $$\begin{array}{l} ROUGE-N=\frac{\underset{S\in Ref}{\sum }\underset{{gram}_{n}\in S}{\sum }{Count}_{match}\left({gram}_{n}\right)}{\underset{S\in Ref}{\sum }\underset{{gram}_{n}\in S}{\sum }\text{Count}\left({gram}_{n}\right)} \end{array}$$

In Equation (6), *n* represents the length of n-grams, *Ref* is the set of reference summaries. *Count*_match_(*gram*_*n*_) is the maximum number of n-grams that appear simultaneously in the generated summary and the corresponding reference summary, while *Count*(*gram*_*n*_) is the number of n-grams in the reference summary. The calculation formula for ROUGE-L is described by Equations (7–9).


(7) $$\begin{array}{l} R_{\text{LCS}}=\frac{LCS\left(C,S\right)}{len\left(S\right)} \end{array}$$

(8) $$\begin{array}{l} P_{\text{LCS}}=\frac{LCS\left(C,S\right)}{len\left(C\right)} \end{array}$$

(9) $$\begin{array}{l} F_{\text{LCS}}=\frac{\left(1+\beta^{2}\right)R_{\text{LCS}}P_{\text{LCS}}}{R_{\text{LCS}}+\beta^{2}P_{\text{LCS}}} \end{array}$$

In Equations (7-9), *R*_LCS_ represents recall, *P*_LCS_ represents precision, and *F*_LCS_ denotes the ROUGE-L value. β is a tunable parameter, and in this paper, it is set to 0.5, indicating that *F*_LCS_ gives equal importance to *R*_LCS_ and *P*_LCS_.

Due to the specificity of the ATC domain, repetition must be completely accurate to be considered a valid repetition instruction. Pilot repetition instructions require responding to the control instructions based on ATC rules without losing any crucial information. According to ATC rules (Drayton and Coxhead, 2023), ATCO instructions must start with the aircraft identification (ACID) to specify the communicating aircraft, while pilot repetitions should end with their ACID to differentiate them from ATCO instructions. Based on the characteristics of the generated repetitions mentioned above, using only the ROUGE evaluation metric cannot comprehensively assess the model's performance. For example, in the control instruction dataset, the controller issues the Chinese control instruction “MU5424, yi jing xiang Beijing shen qing, xian zan shi bao chi 7500”, and the reference repetition instruction is “Yi jing xiang Beijing shen qing, xian zan shi bao chi 7500, MU5424”. After word segmentation, the tokens are as follows: “Yi jing/xiang/Beijing/shen qing/zan shi/bao chi/7500/MU5424”. When the model generates the result “Zan shi/bao chi/7500/MU5424”, evaluating the result using the ROUGE-N and ROUGE-L evaluation methods yields the results shown in Table 1. However, from the perspective of repetition generation rules, this repetition instruction is correct.

**Table 1:** Calculation results of ROUGE-1, ROUGE-2, and ROUGE-L for the example.

**Evaluation metrics**	**Number of *n*-grams in the reference instruction**	**Number of overlapping *n*-grams between the repetition and the reference**	**Result**
ROUGE-1	8	4	0.5
ROUGE-2	7	3	0.429
ROUGE-L	8	4	0.5

From the results in Table 1, it can be seen that although the ROUGE metrics can to a large extent reflect the quality of the generated repetition instructions, there are times when unreasonable situations may arise. Therefore, considering the characteristics of ATC instructions and the repetition criteria, we introduce a new evaluation metric specific to this domain, based on keyword evaluation. The evaluation metrics include Call Sign Accuracy (CSA), Action Instruction Accuracy (AIA), and Parameter Accuracy (PA). Finally, the Total Accuracy (TA) is calculated. Only when an instruction has all three sub-factors correctly, it can be considered as a correct repetition instruction. The definitions and calculation formulas of the specific metrics are as follows: (1) Call sign is composed of the airline abbreviation and flight number, and its accuracy is calculated using the following formula.


(10) $$\begin{array}{l} CSA=\frac{1}{N}\overset{N}{\underset{i=1}{\sum }}g\left(i\right) \end{array}$$

(2) Action instruction refers to the actions contained in the ATC instruction, such as climb, descend, maintain, etc., and its accuracy is calculated using the following formula.


(11) $$\begin{array}{l} AIA=\frac{1}{N}\overset{N}{\underset{i=1}{\sum }}q\left(i\right) \end{array}$$

(3) Parameter refers to the key supplementary information of the instruction actions in the ATC instruction, including speed, altitude, heading, waypoints, etc., and its accuracy is calculated using the following formula.


(12) $$\begin{array}{l} PA=\frac{1}{N}\overset{N}{\underset{i=1}{\sum }}h\left(i\right) \end{array}$$

In Equations (10–12), *N* represents the number of samples to be tested, and *g*(*i*), *q*(*i*), and *h*(*i*) represent the feature functions of call sign, action instruction, and parameter of the instruction, respectively. The specific formulas is described by Equation (13).


(13) $$\begin{array}{l} \begin{array}{c} g\left(i\right),q\left(i\right),h\left(i\right)=\{\begin{array}{cc} 1 & if\;{pred}_{i}={truth}_{i}\\ 0 & otherwise \end{array} \end{array} \end{array}$$

(4) TA represents the total accuracy, which is the sentence-level accuracy. A generated repetition is considered valid and correct only when the call sign, parameters, and action instructions in the repetition match the ground truth. The specific formulas are described by Equations (14, 15).


(14) $$\begin{array}{l} T\left(i\right)=\{\begin{array}{cc} 1 & if\;g\left(i\right)=q\left(i\right)=h\left(i\right)\\ 0 & otherwise \end{array} \end{array}$$

(15) $$\begin{array}{l} TA=\frac{1}{N}\overset{N}{\underset{i=1}{\sum }}T\left(i\right) \end{array}$$

In Equation (15), *N* represents the number of samples to be tested, *T*(*i*) is the feature function for total accuracy.

### 4.4. ATC Corpus Segmentation Dictionary

To facilitate the ROUGE evaluation and keyword evaluation of repetition instructions, we built a Chinese Air-Ground Communication Segmentation Dictionary based on the training data and reference the regulation “Radio Communication Phraseology for Air Traffic Services” (MH/T 4014-2003), as well as the abbreviation standards. We used the Jieba segmentation tool to construct the dictionary, which includes aviation company abbreviations, numbers, letters, altitude levels, speeds, headings, waypoints, proper nouns, and other relevant terms. The dictionary consists of a total of 14,756 vocabulary entries. A sample analysis of the vocabulary is presented in Table 2.

**Table 2:** Examples of word entries in the dictionary.

**Category**	**Example**
Airline abbreviations	Air China, Eastern, CA, MU, Sichuan, 3U, etc.
Numbers	0 (“dong”), 1 (“yao”), 2 (“liang”), 7 (“guai”), etc.
Altitude	600, 900, 1,200, 1,500, …, 13,700
Speed	250 knots, 180 knots, etc.
Heading	Direct flight, offset, flying heading, etc.
Waypoint	Dawangzhuang, BUBDA, ANDIN, P23, etc.
Proper noun	Indicated airspeed, field pressure, planned route, instrument flight, etc.

## 5. Experiments and discussions

### 5.1. Dataset

**Figure 4:**
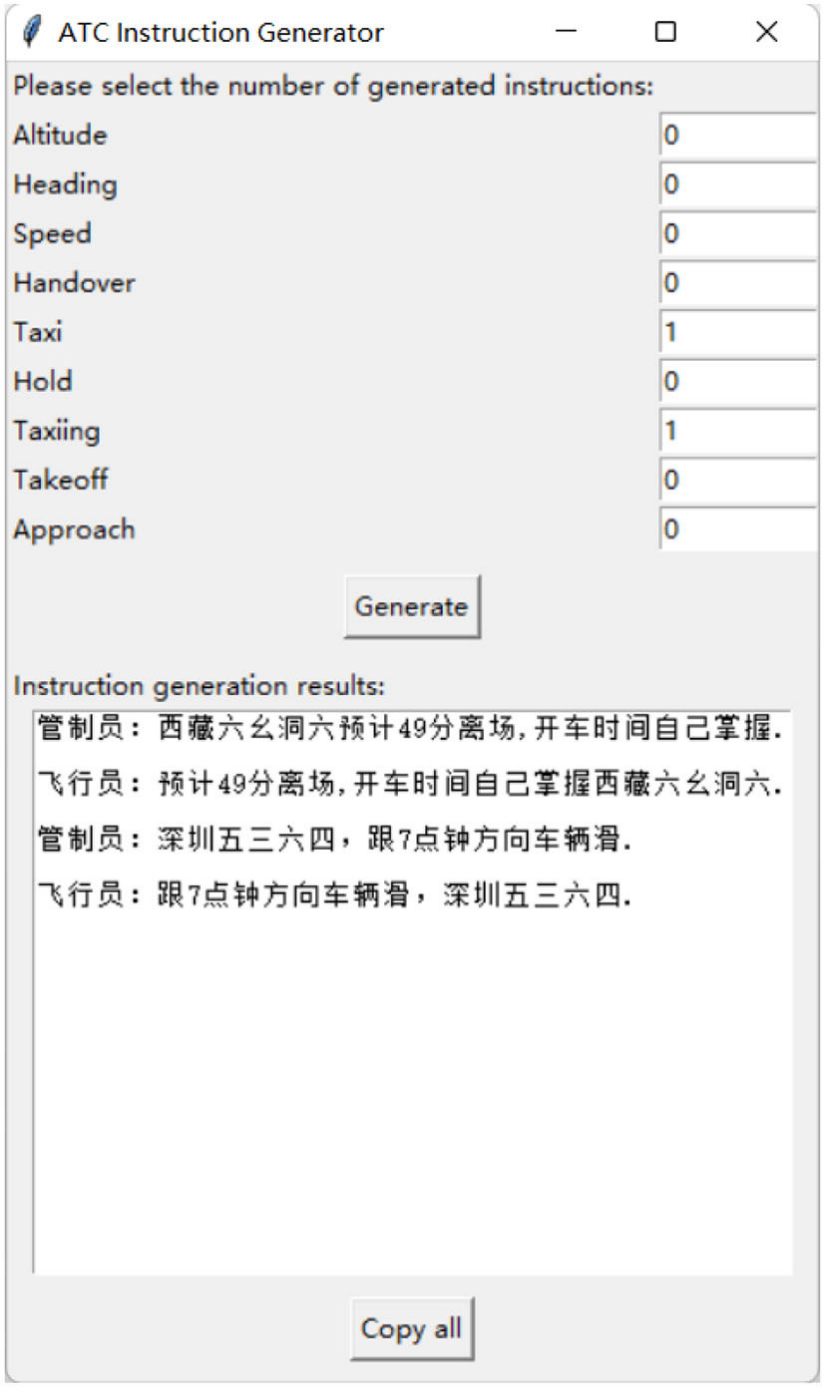
UI Interface of the tower control instruction generator.

**Figure 5:**
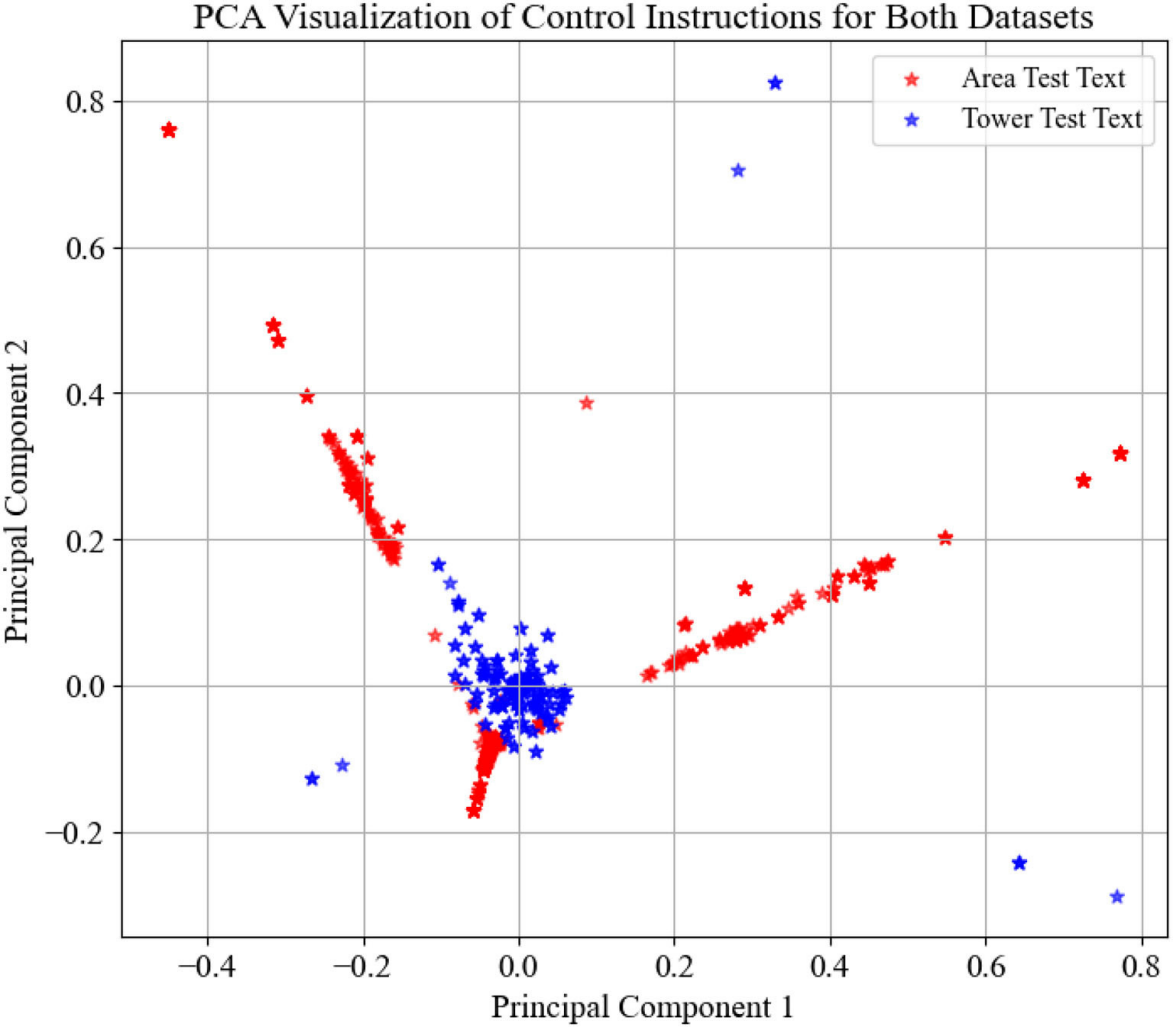
Distribution of tower control dataset and area control dataset.

The experiment consists of two datasets: the area control dataset and the tower control dataset. The area control dataset comprises real air-to-ground communication data in actual ATC scenarios. The tower control dataset, on the other hand, is generated by computer based on the standards, and its User Interface (UI) is shown in Figure 4. You can find this algorithm in this link https://drive.google.com/drive/folders/1RN6CEhJXcoru6LyZB8u_Y3XBLjyvlQqd?usp=sharing. To illustrate the distribution of these two datasets, we utilized Term Frequency-Inverse Document Frequency (TF-IDF) for data vectorization and employed Principal Component Analysis (PCA) for dimensionality reduction to achieve data visualization. The dataset distributions are depicted in Figure 5.

In Figure 5, the distribution represented by red stars corresponds to the area control dataset, while the distribution denoted by blue stars corresponds to the tower control dataset. It is evident that the tower control dataset encompasses a significantly different set of instruction types compared to the area control dataset, which can be used to assess the model's generalization capability.

The dataset for training the area control consists of 11,049 pairs, with 8,949 pairs used for training, 995 pairs for validation, and 1,105 pairs for testing. The tower control dataset, used for transfer learning generalization evaluation, contains a total of 1,074 pairs. Table 3 displays some examples from the dataset.

**Table 3:** Dataset example table.

**Dataset name**	**Control instructions**	**Pilot recitation instructions**
Tower	Jinxiu 7443, estimated departure time 10 min.	Estimated departure time is 10 min, Jinxiu 7443.
	Hebei 8554, circling and waiting over JHG.	Circling and waiting over JHG, Hebei 8554.
Area	Shandong 8896, Xiamen, radar has been identified.	Radar has identified, Shandong 8896.
	Hainan 7064, cancel offset return route.	Cancel offset return route, Hainan 7064.

### 5.2. Experiment configurations

The experiments were conducted on a Windows operating system. The computer configuration is as follows: Intel Core i5-8400 processor, 56 GB of RAM, NVIDIA RTX 4090 24 GB graphics card, 250 GB SSD, and a 3.6 TB HDD. The deep learning framework used was PyTorch. The hyperparameters for the RoBERTa-RL model are listed in Table 4.

**Table 4:** Hyperparameters for the RoBERTa model.

**Hyperparameter**	**Setting**
Dropout	0.1
Max sequence length	256
Learning rate	0.0001
Batch size	32
Number of epochs	20
Optimizer	Adam
Beamsearch size	3
Weight decay	0.001
λ	0.5

### 5.3. Ablation experiment

To demonstrate the effectiveness of the adopted strategies, we conducted ablation experiments for validation, using ROUGE-N and ROUGE-L as evaluation metrics. The experimental results are shown in Table 5.

**Table 5:** Experimental results based on ROUGE evaluation metrics.

**Model**	**Dataset**	**ROUGE-1**	**ROUGE-2**	**ROUGE-L**
RoBERTa-RL (λ = 0)	Area	0.995	0.990	0.994
	Tower	0.885	0.704	0.885
RoBERTa-RL (λ = 0.3)	Area	0.996	0.991	0.995
	Tower	0.980	0.946	0.980
**RoBERTa-RL (λ** **= 0.5)**	**Area**	**0.996**	**0.991**	**0.995**
	**Tower**	**0.982**	**0.954**	**0.982**
RoBERTa-RL (λ = 1.0)	Area	0	0	0
	Tower	0	0	0

The meaning of the bold values is the optimal values achieved by the RoBERTa-RL (λ = 0.5) model across different datasets and metrics.

According to Table 5, it can be observed that RoBERTa-RL(λ = 0), the unimproved RoBERTa model, achieves good performance on the area control dataset through transfer learning. However, it performs poorly on the tower control dataset, indicating a problem of poor generalization when relying solely on transfer learning. When λ = 0.3, it can be seen that the model has overcome the issue of poor generalization and shows further improvement compared to λ = 0. When λ = 0.5, the model reaches optimal performance. This is because choosing a reward weight of 0.3 emphasizes the cross-entropy loss. On the other hand, a reward weight of 0.5 balances the contribution of the cross-entropy loss and the reward function. This setting can to some extent balance the quality and grammatical accuracy of the generated instructions, leading to better performance. Setting the reward weight λ to 1, without considering the cross-entropy loss, means only optimizing the similarity between the generated results and the reference summaries, without considering grammatical accuracy and the optimization of the generation strategy. This results in the model disregarding grammar rules and sentence structure during the generation process, leading to the generation of unreasonable instructions.

### 5.4. Contrastive experiments

**Figure 6:**
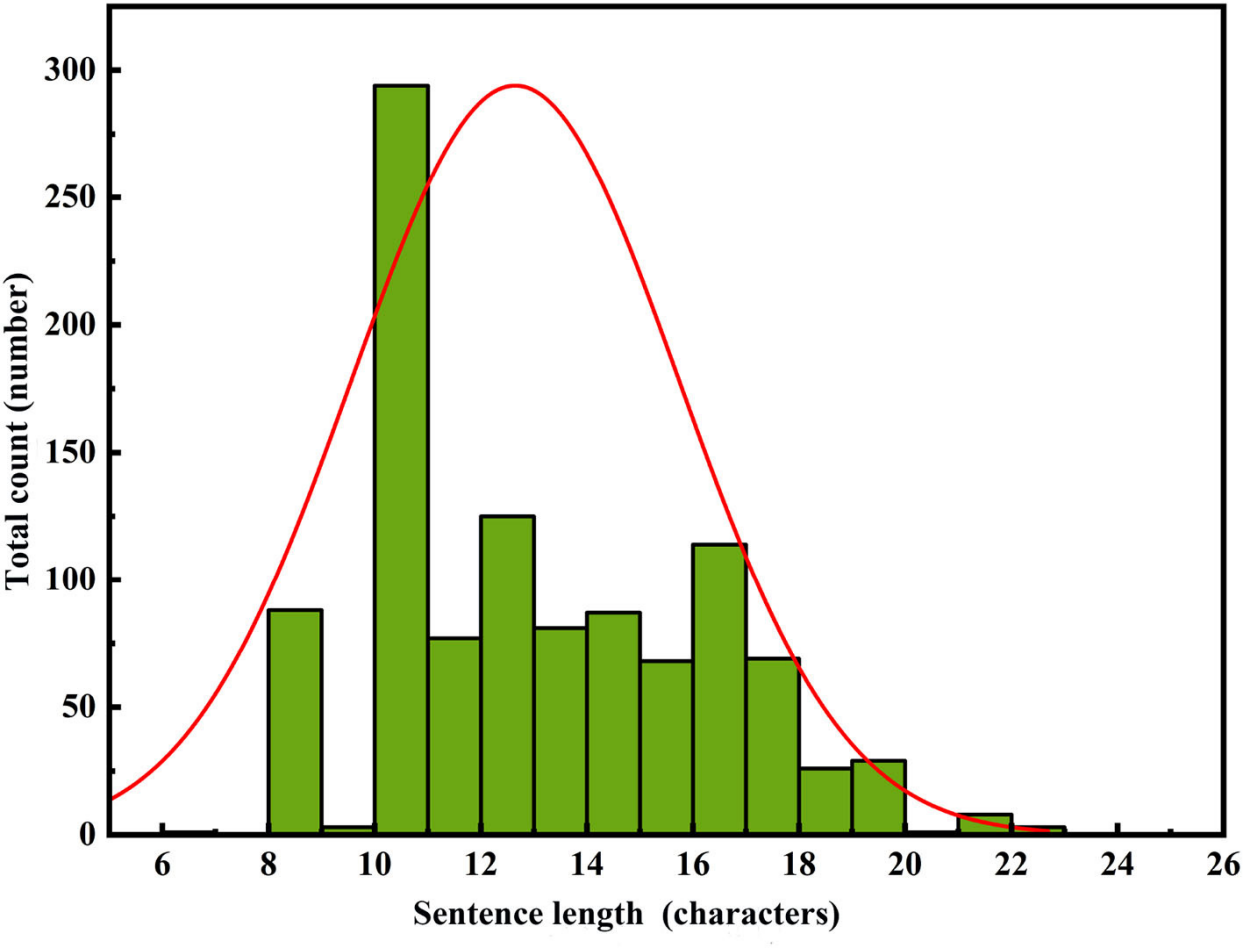
RoBERTa-RL (λ = 0) PRG text length distribution.

**Figure 7:**
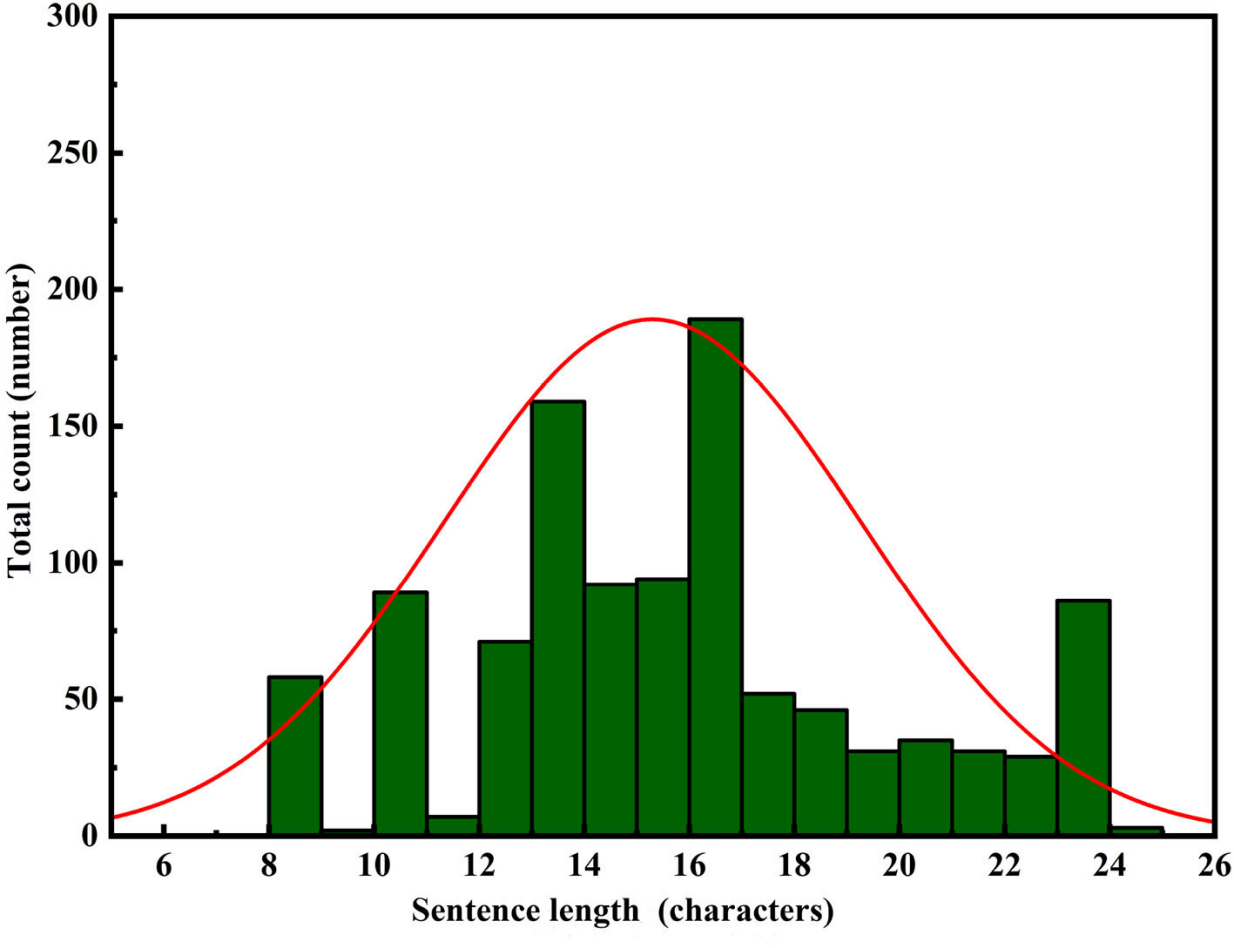
Reference label length distribution.

**Figure 8:**
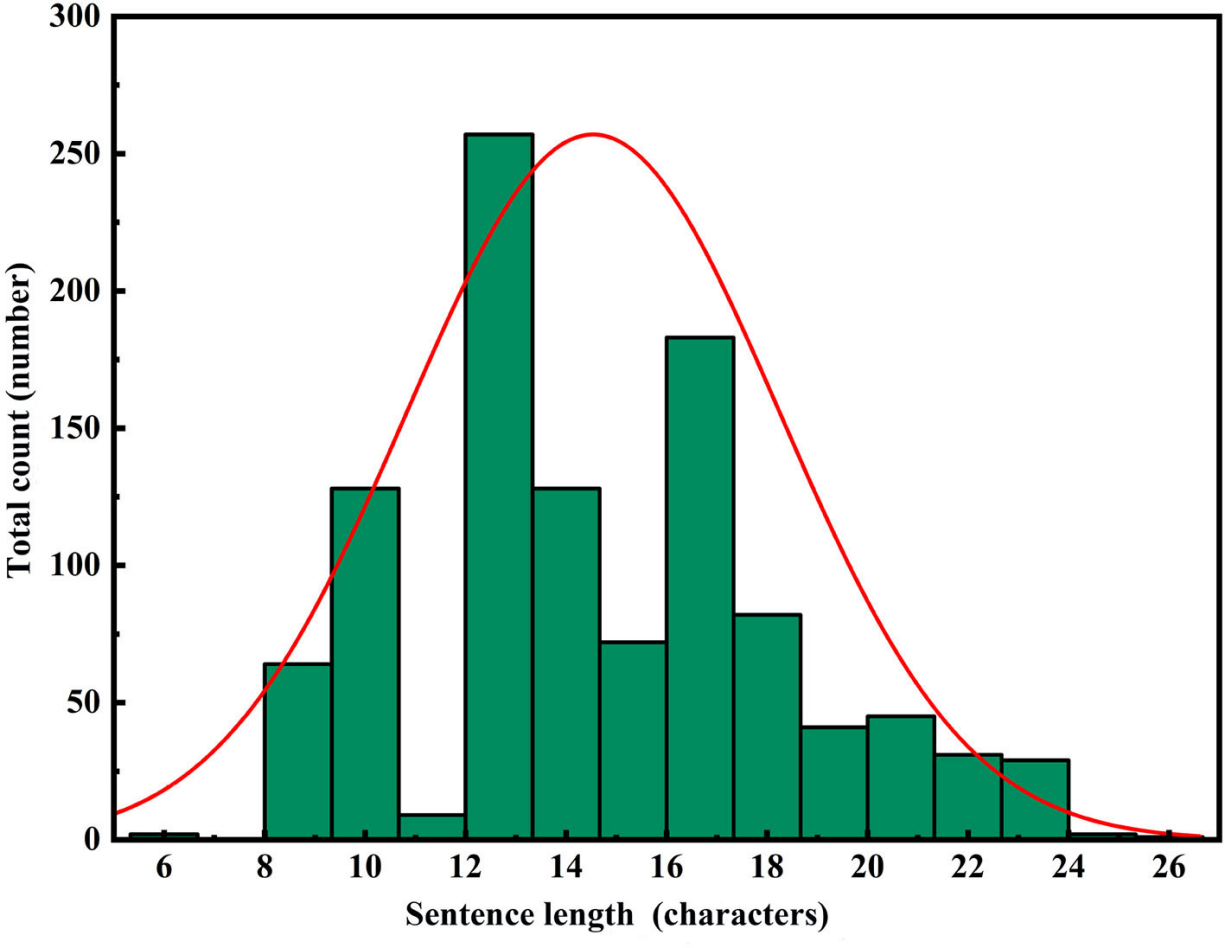
RoBERTa-RL (λ=0.5) PRG text length distribution.

To perform a comprehensive analysis of the constructed model's performance, we adopted a comparative research approach tailored to the application domain. Specifically, we evaluated the performance of the constructed model as well as leading pre-trained models in the field of text generation, namely GPT-2, BERT, and BART, in the task of repetition instruction generation. Tests were conducted separately on the area control dataset and the tower control dataset, with evaluation metrics including ROUGE-N, ROUGE-L, and keyword evaluation criteria. The experimental results are presented in Tables 6, 7. Furthermore, to visualize the improvements made by the model, we compiled statistics on the length distribution of repetition instructions generated by the model before and after enhancements on the tower control test dataset. The visual results are illustrated in Figures 6–8.

**Table 6:** Comparative experimental results based on ROUGE evaluation metrics.

**Model**	**Dataset**	**ROUGE-1**	**ROUGE-2**	**ROUGE-L**
GPT2	Area	0.981	0.973	0.981
	Tower	0.779	0.61	0.776
BERT	Area	0.991	0.984	0.991
	Tower	0.846	0.662	0.846
BART	Area	0.992	0.987	0.992
	Tower	0.910	0.767	0.910
RoBERTa-RL (λ = 0)	Area	0.995	0.990	0.994
	Tower	0.885	0.704	0.885
**RoBERTa-RL (λ** **= 0.5)**	**Area**	**0.996**	**0.991**	**0.99**6
	**Tower**	**0.982**	**0.954**	**0.982**

The meaning of the bold values is the optimal values achieved by the RoBERTa-RL (λ = 0.5) model across different datasets and metrics.

**Table 7:** Comparative experimental results based on keyword evaluation metrics.

**Model**	**Dataset**	**CSA (%)**	**AIA (%)**	**PA (%)**	**TA (%)**
GPT2	Area	99.1	98.0	98.5	96.8
	Tower	89.3	81.4	24.5	23.3
**BERT**	Area	99.6	98.8	98.8	97.4
	**Tower**	**100.0**	**99.8**	25.6	25.6
BART	Area	99.2	98.8	98.2	96.8
	Tower	99.0	94.1	35.1	34.3
RoBERTa-RL (λ = 0)	Area	99.7	98.2	99.4	97.6
	Tower	98.7	94.5	25.8	25.8
**RoBERTa-RL (λ** **= 0.5)**	**Area**	**100.0**	**99.1**	**99.5**	**98.8**
	**Tower**	99.7	98.7	**82.5**	**81.8**

The meaning of the bold values is the optimal values achieved by the RoBERTa-RL (λ = 0.5) model across different datasets and metrics.

From Table 6, it can be observed that all comparative models performed well on the area control dataset. The proposed RoBERTa-RL(λ = 0.5) model only slightly outperformed the comparative models. However, on the tower control dataset, all comparative models showed poor generalization performance, while our proposed model's performance only slightly decreased. Table 7 provides a detailed display of the performance of each transfer learning model based on the Keyword Evaluation Metrics. From Table 7, it is visually evident that the comparative models performed poorly on the tower control dataset, indicating a clear issue of poor generalization. Additionally, the GPT-2 model performed the worst in the task, possibly due to its use of masked attention mechanism during prediction, which failed to incorporate useful information from the context. Finally, our constructed RoBERTa-RL(λ = 0.5) model achieved the best performance on the tower control dataset, demonstrating that the proposed improvement strategies greatly alleviate the issue of poor generalization in transfer learning.

In Figures 6–8, the horizontal axis represents the string length of repetition instructions, while the vertical axis denotes the total count of repetition instructions of varying lengths. The red curve illustrates the length distribution of repetition instructions. By comparing Figures 6, 7, we observe that the mean length of repetition instructions generated by RoBERTa-RL (λ = 0) is lower than the mean length of reference labels, indicating a significant omission of words and poor generalization for this model. However, by comparing Figures 7, 8, we can see that the RoBERTa-RL (λ = 0.5) model generates repetition instructions with a length similar to the mean length of reference labels, effectively mitigating the omission issue and demonstrating strong generalization.

In addition, we analyzed the reasons behind the model's strong generalization capability. Specifically, due to the disparities in data distribution between the area control dataset and the tower control dataset, the baseline model fine-tuned on the area control dataset performed poorly on the tower control dataset. This generalization issue is a common challenge faced by most fine-tuned models at the current stage. However, the introduction of reinforcement learning strategies effectively mitigates this problem. During the training process, we incorporated a reward and penalty mechanism to assess the quality of generated results and provide timely feedback to the model. This mechanism encourages the model to prioritize the quality of the generated text over similarity to the target labels, thereby preventing overfitting to the training data distribution. Furthermore, the introduction of the reward and penalty mechanism essentially transforms the model into a multitask learning problem, where one task is to generate repetition instructions, and the other task is to learn how to generate high-quality instructions to maximize rewards. As a result, the model's generated results exhibit strong performance on datasets with different distributions. Finally, setting the weights of both the reinforcement learning loss and the original cross-entropy loss to 0.5 ensures that the model does not overly rely on either aspect during optimization but strikes a balance between the two objectives, thereby enhancing the overall model performance. In summary, reinforcement learning strategies are advantageous in enabling the model to learn deep features of the dataset, allowing the model to excel on similar yet differently distributed datasets. This approach is highly effective and can be applied to many similar problems to improve model generalization capabilities.

## 6. Conclusions

Our research focuses on addressing the problem of generating high-quality pilot recitations in the ATC field based on small-scale training data. To tackle this challenge, we propose a DRL model that optimizes the cross-entropy loss using the policy gradient algorithm to overcome exposure bias and poor generalization in transfer learning. Through a series of experiments, we demonstrate that our proposed model outperforms the comparison models on the training dataset and maintains excellent performance on similar distribution datasets. To expedite model training, we employ a pretraining method based on cross-entropy loss and a training strategy that combines the policy gradient algorithm with cross-entropy loss. This strategy allows the model to converge faster and reduces resource consumption. In addition to the commonly used ROUGE evaluation metric, we introduce a keyword-based evaluation metric to assess the model's performance. The results show that the keyword-based evaluation metric provides a more accurate reflection of the model's performance. On the tower control dataset, our proposed model achieves an overall accuracy of 81.8%, which is a 56% improvement compared to the pre-improved model and a 47.5% improvement compared to the other comparable models.

However, it is essential to consider some potential safety implications that the model may introduce in practical applications. At the current stage, since the model's input is limited to textual information alone, it lacks sufficient contextual information to assess the reasonableness of the control instructions it receives. As a result, it cannot generate queries or doubts about control instructions that could lead to flight conflicts. To facilitate the deployment of the model in real-world scenarios, it is imperative that the model, in addition to processing text data, can also incorporate navigation and monitoring data. In our future work, we will integrate these multimodal data sources as inputs to the repetition generation model, enabling it to scrutinize and question conflicting or unreasonable control instructions, thereby further mitigating safety risks.

## Data availability statement

The datasets presented in this study can be found in online repositories. The names of the repository/repositories and accession number(s) can be found below: https://drive.google.com/drive/folders/1RN6CEhJXcoru6LyZB8u_Y3XBLjyvlQqd?usp=sharing.

## Author contributions

WP: Writing—review and editing. PJ: Writing—original draft. YL: Writing—review and editing. ZW: Writing—review and editing. JH: Writing—review and editing.
